# The complete mitochondrial genome sequence of *Rana dabieshanensis* (Anura: Ranidae)

**DOI:** 10.1080/23802359.2021.1920502

**Published:** 2021-07-19

**Authors:** Jingmei Gan, Ying Li, Yaqiong Wan, Jiaqi Li, Baowei Zhang

**Affiliations:** a School of Life Sciences, Anhui University, Hefei, China; b Environmental Protection Key Laboratory on Biosafety, Nanjing Institute of Environmental Science, Ministry of Ecology and Environment, Nanjing, Jiangsu, China

**Keywords:** *Rana dabieshanensis*, mitochondrial genome, phylogenetic analysis

## Abstract

*Rana dabieshanensis* is a species of frog within the family Ranidae. In this study, we assembled a complete mitochondrial genome (mito-genome) for *R. dabieshanensis* by high-throughput sequencing technology. It is 18,291 bp and includes 13 protein-coding genes, 22 tRNA genes, two rRNA genes and one control region. The nucleotide composition is A: 27.0%, T: 28.6%, C: 29.3% and G: 15.2%. Two overlaps among the 13 protein-coding genes were found: *ATP8*/*ATP6*, *ND4L*/*ND4*. The study of phylogenetic analysis based on complete mitochondrial genome showed that there was close genetic relationship between *R. dabieshanensis* and *R. omeimontis* and it is useful for systematic analyses of genus *Rana*.

The Dabie mountain brown frog (*Rana dabieshanensis*) belongs to the family Ranidae, which is distributed in Dabie Mountains regions, Anhui Province, China (Wang et al. 2017). It is controversy about the classification of species within the genus *Rana* in the Dabie Mountains (Wang 2018). To better understand the mitochondrial genomic characteristics, phylogeny and evolution of the *R. dabieshanensis*, we determined and described the mitogenome sequence of *R. dabieshanensis* in order to obtain basic genetic information about this species.

A specimen of *R. dabieshanensis* was collected from Sucheng County, Anhui Province, China (31°31′13.61″N, 116°32′44.87″E) and stored in Anhui University Museum, Research Center for Biology (Voucher number: DBS202001). The genomic DNA extraction, library preparation and Illumina sequencing were done by Novogene Bioinformatics Technology Co. Ltd. (Tianjin, China).

We obtained the complete mitochondrial genome of *R. dabieshanensis* is 18,291 bp and submitted to the GenBank with the accession number MW526989. It included 13 protein-coding genes, two ribosomal RNAs genes, 22 transfer RNAs genes and one control region. The overall nucleotide composition is A: 27.0%, T: 28.6%, C: 29.3% and G: 15.2%, with a total A + T content of 55.6%. The mitogenome of *R. dabieshanensis* shows the typical gene observed in Ranidae mitogenomes (Liu et al. 2017; Fang et al. 2020; Jiang et al. 2020). Within 37 mitochondrial genes, the *ND6* gene and 8 tRNA genes (*tRNA^Ser^*, *tRNA^Glu^*, *tRNA^Pro^*, *tRNA^Gln^*, *tRNA^Ala^*, *tRNA^Asn^*, *tRNA^Cys^* and *tRNA^Tyr^*) were encoded on the light strand and other genes were encoded by the H-strand. In 13 mitochondrial protein-coding genes, except *COX1*, *ATP6* and *ND4L* begin with GTG, *ND6* begin with TCT, the other nine genes begin with ATG as start codon. In all six types of stop codon were annotated, TAT for *ND2*, AGG for *COX1* and *ND5*, TAG for *ATP8*, TAA for *ND4L* and *CYTB*, CAT for *ND6* and an incomplete stop codon T for the remaining six PCGs (*COX2*, *ATP6*, *COX3*, *ND3*, *ND4* and *ND1*), which is presumably completed as TAA by posttranscriptional polyadenylation (Ojala et al. 1981). With regard to the codon of *ND6* and *ND2*, it is consistent with the *Rana omeimontis* submitted on NCBI (Jiang et al. 2020). Two overlaps among the 13 protein-coding genes were found: *ATP8*/*ATP6* and *ND4L*/*ND4*. The two rRNA genes were 930 bp (*rrnS*) and 1576 bp (*rrnL*), respectively.

To best understand its phylogenetic position within the genus *Rana*, we reconstructed the phylogenetic tree of some species in *Rana* with maximum-likelihood method (ML) (http://iqtree.cibiv.univie.ac.at/), which is based on 13 complete mitochondrial genome sequences for *Rana* species. Another species (*Pelophylax nigromaculatus*) is included as the outgroup (Figure 1). The result showed that phylogenetic relationship of Dabie mountains wood frog is very close to *R. omeimontis*.

**Figure 1.**
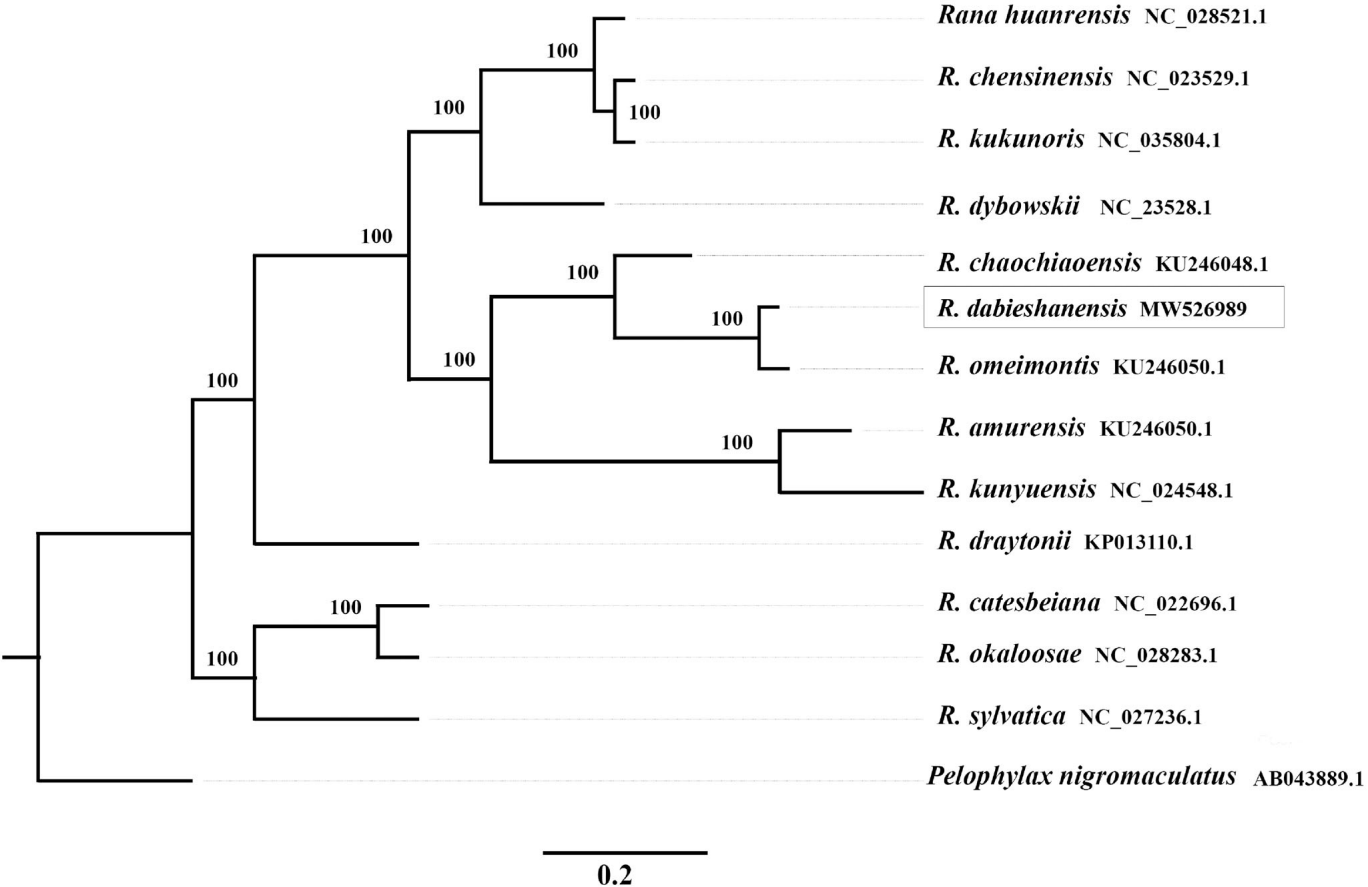
Phylogenetic tree of the relationships of *Rana dabieshanensis* with maximum-likelihood method (ML) based on complete mitochondrial sequences. The bootstrap support values for ML analyses are shown orderly on the nodes.

## Data Availability

The data that support the findings of this study are openly available in NCBI at https://www.ncbi.nlm.nih.gov/ (MW526989).
